# Cadmium phosphates Cd_2_(PO_4_)OH and Cd_5_(PO_4_)_2_(OH)_4_ crystallizing in mineral structures

**DOI:** 10.1107/S2056989024000793

**Published:** 2024-01-26

**Authors:** Felix Eder, Matthias Weil

**Affiliations:** a TU Wien, Institute for Chemical Technologies and Analytics, Division of Structural Chemistry, Getreidemarkt 9/E164-05-01, 1060 Vienna, Austria; University of Neuchâtel, Switzerland

**Keywords:** crystal structure, cadmium phosphate(V), triplite, arsenoclasite, isotypism, structure comparison

## Abstract

Single crystals of two basic cadmium phosphates, dicadmium orthophosphate hydroxide, Cd_2_(PO_4_)OH, and penta­cadmium bis­(orthophosphate) tetra­kis­(hydroxide), Cd_5_(PO_4_)_2_(OH)_4_, were obtained under hydro­thermal conditions. Cd_2_(PO_4_)OH adopts the triplite [(Mn,Fe)_2_(PO_4_)F] structure type. Its asymmetric unit comprises two Cd, one P and five O sites, all situated at the general Wyckoff position 8 *f* of space group *I*2/*a*; two of the O atoms are positionally disordered over two sites, and the H atom could not be localized. Disregarding the disorder, distorted [CdO_6_] polyhedra form a tri-periodic network by edge-sharing with neighbouring [CdO_6_] units and by vertex-sharing with [PO_4_] units. The site associated with the OH group is coordinated by four Cd atoms in a distorted tetra­hedral manner forming ^1^_∞_[(OH)Cd_4/2_] chains parallel to [001]. The oxygen environment around the OH site suggests multiple acceptor atoms for possible O—H⋯O hydrogen-bonding inter­actions and is the putative reason for the disorder. Cd_5_(PO_4_)_2_(OH)_4_ adopts the arsenoclasite [Mn_5_(AsO_4_)_2_(OH)_4_] structure type. Its asymmetric unit comprises five Cd, two P, and twelve O sites all located at the general Wyckoff position 4 *a* of space group *P*2_1_2_1_2_1_; the H atoms could not be localized. The crystal structure of Cd_5_(PO_4_)_2_(OH)_4_ can be subdivided into two main sub-units. One consists of three edge-sharing [CdO_6_] octa­hedra, and the other of two edge- and vertex-sharing [CdO_6_] octa­hedra. Each sub-unit forms corrugated ribbons extending parallel to [100]. The two types of ribbons are linked into the tri-periodic arrangement through vertex-sharing and through common [PO_4_] tetra­hedra. Qu­anti­tative structure comparisons are made with isotypic *M*_5_(*X*O_4_)_2_(OH)_4_ crystal structures (*M* = Cd, Mn, Co; *X* = P, As, V).

## Abstract 2

Cd_2_(PO_4_)OH and Cd_5_(PO_4_)_2_(OH)_4_ crystallize in the mineral structures of triplite and arsenoclasite, respectively.

## 1. Chemical context

In the quest for new oxidotellurates(IV) modified by incorporation of tetra­hedral phosphate anions (Eder & Weil, 2020 ▸; Ok & Halasyamani, 2006 ▸; Yao *et al.*, 2021 ▸; Zhao *et al.*, 2021*a* ▸,*b* ▸; Zimmermann *et al.*, 2011 ▸), crystals of Cd_2_(PO_4_)OH were serendipitously obtained under hydro­thermal conditions when working in the system Cd/Te^IV^/P^V^/O/(H). During a targeted synthesis of Cd_2_(PO_4_)OH under Te-free conditions, another phosphate with composition Cd_5_(PO_4_)_2_(OH)_4_ had crystallized. We report here the synthesis conditions and crystal structure refinements of these two basic cadmium phosphates and their relationships with known mineral structures.

## 2. Structural commentary

So far, structural data for basic cadmium phosphates have only been reported for apatite-type Cd_5_(PO_4_)_3_(OH) (Hata *et al.*, 1978 ▸). The title compounds, which are described here for the first time, crystallize with known mineral structures. Cd_2_(PO_4_)(OH) adopts the triplite structure, which was first reported by Waldrop (1968 ▸). Triplite is a mineral with composition (Fe,Mn)_2_(PO_4_)F, and other natural and synthetic compounds with the composition *M*_2_(*X*O_4_)*Y* share this structure type, where *M* = Mn, Fe, Cd, Co, Mg; *X* = P, As; *Y* = F, (F,OH) (Đorđević & Kolitsch, 2013 ▸). Cd_5_(PO_4_)_2_(OH)_4_ crystallizes isotypically with the mineral arsenoclasite [Mn_5_(AsO_4_)_2_(OH)_4_], the crystal structure of which was determined by Moore & Molin-Case (1971 ▸). Other isostructural compounds are synthetic Cd_5_(VO_4_)_2_(OH)_4_ (Karanović & Đorđević, 2022 ▸), Co_5_(PO_4_)_2_(OH)_4_ and Mn_5_(PO_4_)_2_(OH)_4_ (Ruszala *et al.*, 1977 ▸), as well as the natural variant of Mn_5_(PO_4_)_2_(OH)_4_ – the mineral gatehouseite (Elliott & Pring, 2011 ▸).

Cd_2_(PO_4_)OH is the first reported *M*_2_(*X*O_4_)*Y* compound with exclusively OH^−^ ions at the *Y* site to crystallize in the triplite structure in space-group type *I*2/*a*. Such *M*_2_(*X*O_4_)OH compounds usually adopt the triploidite structure in space-group type *P*2_1_/*a*, like the arsenate analogue Cd_2_(AsO_4_)(OH) (Đorđević & Kolitsch, 2013 ▸). Triploidite-like structures have twice the unit-cell volume of triplite-like structures and show no centering of the monoclinic unit-cell. However, for Cd_2_(PO_4_)OH, reflections hinting at a doubled unit-cell volume or violating the reflection conditions for an *I*-centered unit-cell were not found in the diffraction data.

**Figure 1:**
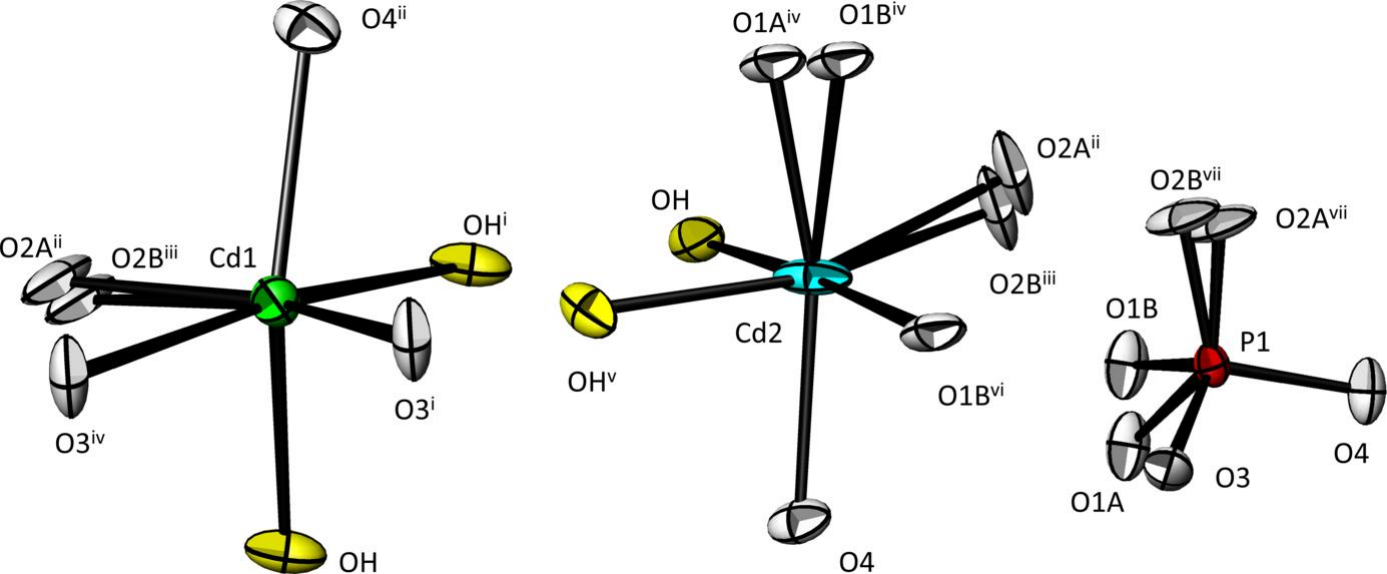
Coordination polyhedra in the crystal structure of Cd_2_(PO_4_)OH showing the disordered atoms O1 and O2. Displacement ellipsoids are drawn at the 50% probability level. Symmetry codes refer to Table 1 ▸.

**Figure 2:**
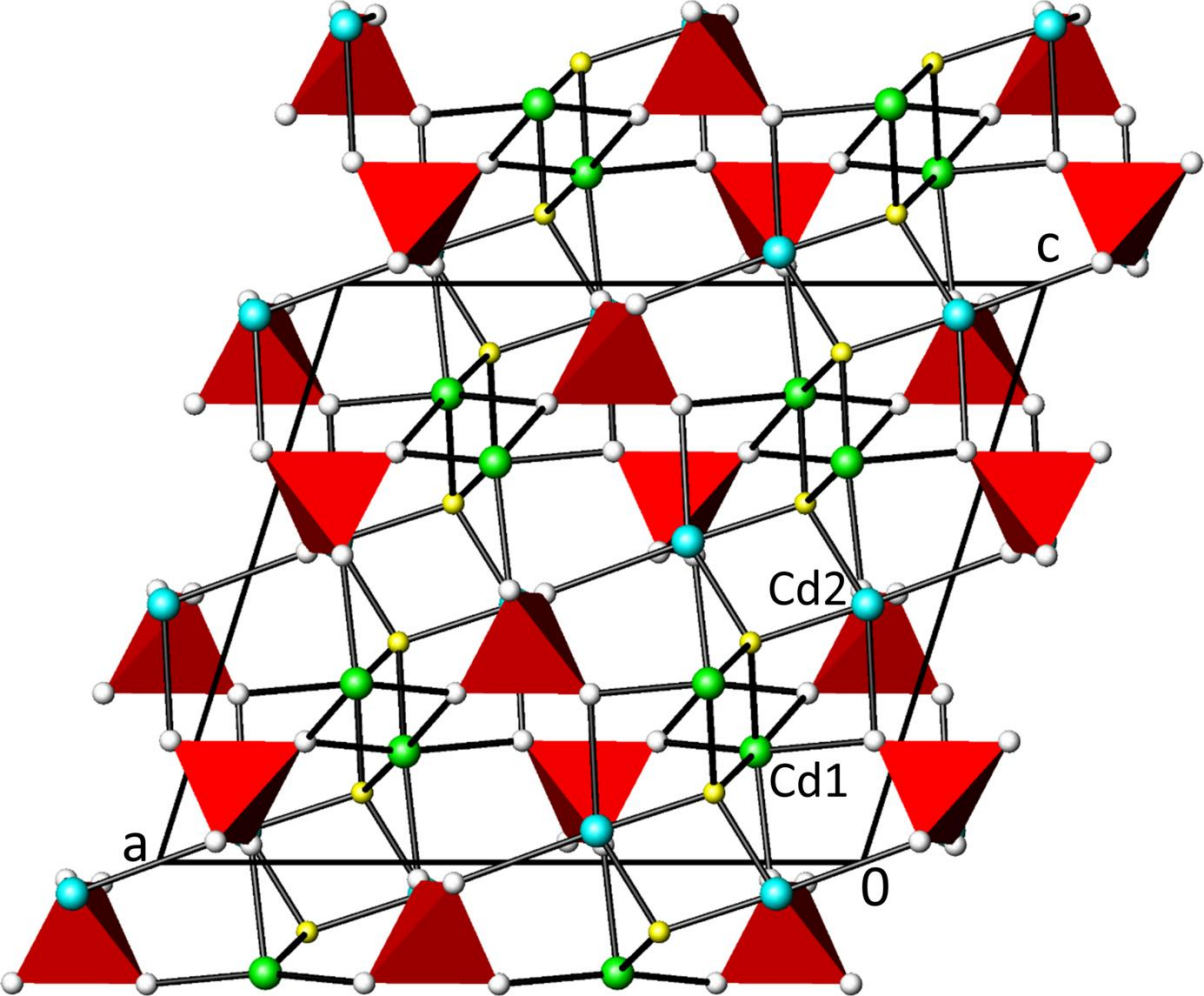
The idealized crystal structure of Cd_2_(PO_4_)OH without disorder of O1 and O2 in a projection along [0 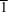 0]. The O atom of the OH group is given as a yellow sphere, the other O atoms as white spheres, the Cd atoms as green spheres; the [PO_4_] unit is displayed as a red polyhedron.

**Cd_2_(PO_4_)OH**. The asymmetric unit of Cd_2_(PO_4_)OH comprises two Cd, one P and five O sites (O1 and O2 being positionally disordered over two sites each). All atomic sites are situated at the general Wyckoff position 8 *f* of space group *I*2/*a.* The resulting coordination polyhedra around Cd1, Cd2 and P are depicted in Fig. 1 ▸. For the sake of simplicity, the crystal structure of Cd_2_(PO_4_)OH will be described in the following without the disorder of atoms O1 and O2. Considering Cd—O distances < 3.0 Å as relevant, both Cd sites are coordinated by six oxygen atoms forming significantly distorted [CdO_4_(OH)_2_] octa­hedra (Table 1 ▸, where only the bonds for O atoms with major occupancy are indicated). The mean Cd—O distances in the two polyhedra (Cd1—O = 2.31, Cd2—O = 2.28 Å) are in good agreement with the literature value of 2.302 (69) Å for six-coordinate Cd (Gagné & Hawthorne, 2020 ▸). Each [CdO_4_(OH)_2_] octa­hedron shares four of its edges with neighbouring [CdO_4_(OH)_2_] octa­hedra, two with each Cd (Cd1 and Cd2). Additionally, each [CdO_4_(OH)_2_] unit shares four of its O atoms with [PO_4_] tetra­hedra, leading to a tri-periodic structure (Fig. 2 ▸).

Of the five oxygen sites, four (O1–O4) are bound to two Cd and one P atom each. The site associated with the OH group is bound to four Cd sites. This assignment is supported by bond-valence calculations (Brown, 2002 ▸), using the parameters of Brese & O’Keeffe (1991 ▸). The bond-valence sum (BVS) of the OH site amounts to 1.67 valence units (1.92–2.08 valence units for the other O sites). The OH site is the one occupied by the F^−^ anion in the isotypic triplite-type structures. The [(OH)Cd_4_] polyhedron has a distorted tetra­hedral shape with bond lengths in the range 2.101 (6)–2.484 (7) Å. The [(OH)Cd_4_] tetra­hedra are linked to each other by sharing two edges with neighbouring tetra­hedra forming ^1^_∞_[(OH)Cd_4/2_] chains extending parallel to [001] (Fig. 2 ▸).

**Figure 3:**
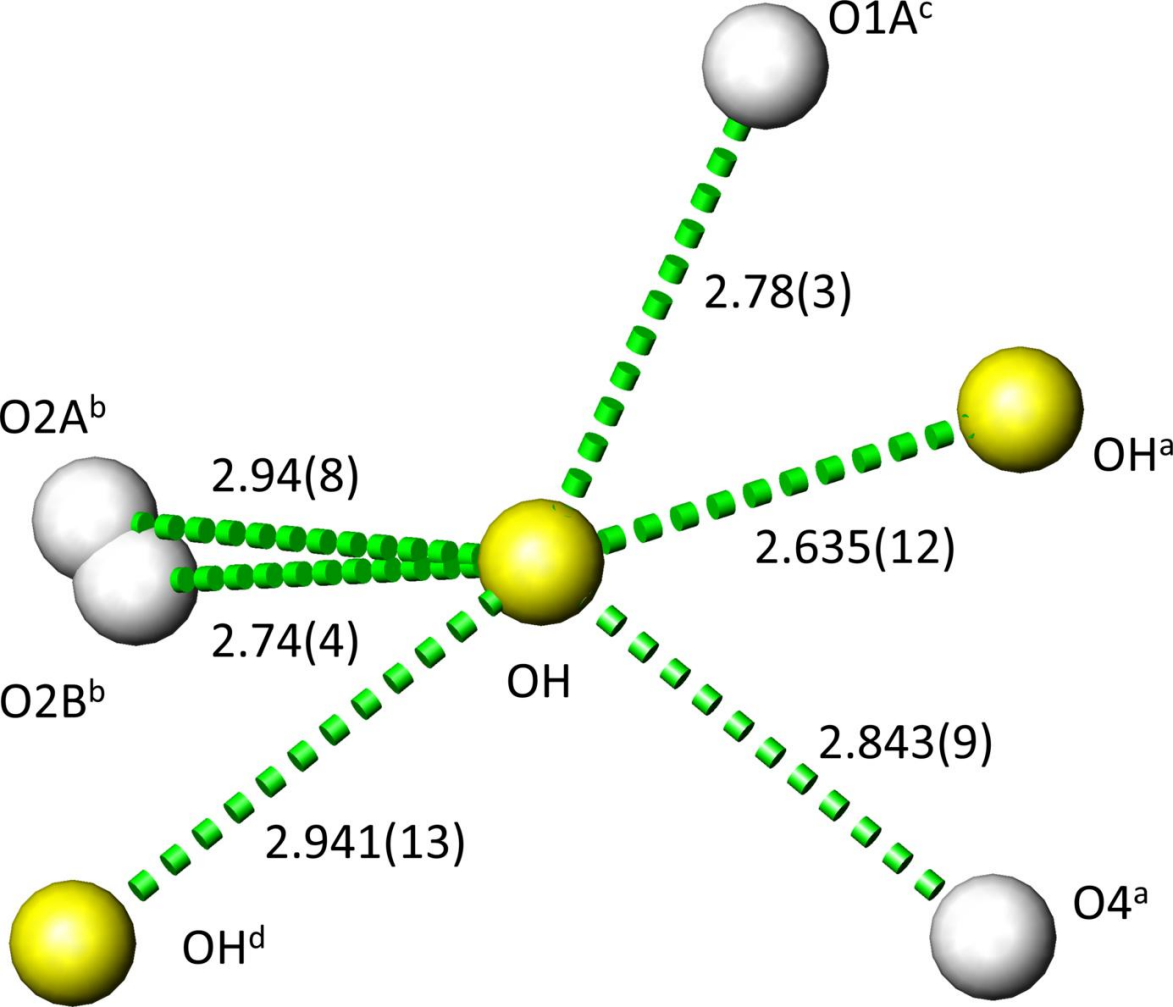
Cd_2_(PO_4_)OH. Environment of the OH site suitable for hydrogen-bonding inter­actions [*d*(OH⋯O) < 3.0 Å]; distances (Å) are indicated. Symmetry codes: (*a*) −*x* + 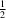 , *y*, −*z* + 1; (*b*) −*x* + 1, *y* − 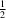 , −*z* + 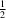 ; (*c*) −*x* + 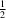 , *y* − 1, −*z* + 1; (*d*) −*x* + 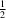 , −*y* + 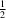 , −*z* + 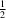 .

The environment of the OH site suggests multiple acceptor atoms for possible O—H⋯O hydrogen-bonding inter­actions and is the putative reason why the hydrogen atom could not be localized and also for the disorder of O1 and O2. Taking into account hydrogen-bonding inter­actions with O⋯O distances < 3.0 Å as significant, there are six O atoms in the vicinity of each OH site (Fig. 3 ▸). The shortest contact amounts to 2.635 (12) Å towards a symmetry-related OH site, the longest to 2.94 (8) Å to O2*A*. In the isotypic crystal structure of Cd_2_(PO_4_)F (Rea & Kostiner, 1974 ▸), the F site (corresponding to the OH site in the title structure) is not split and has four contacts < 3.0 Å to two F sites [2.756 (6) and 2.800 (7) Å] and to two O sites [2.828 (5) and 2.832 (5) Å].

Owing to the disorder present in Cd_2_(PO_4_)OH, a qu­anti­tative comparison with the ordered isotypic *M*_2_(*X*O_4_)*Y* crystal structures adopting the triplite-structure type was not undertaken.

**Cd_5_(PO_4_)_2_(OH)_4_**. The asymmetric unit of Cd_5_(PO_4_)_2_(OH)_4_ comprises five Cd, two P and twelve O sites, all located on the general Wyckoff position 4 *a* of space group *P*2_1_2_1_2_1_; the H-atom sites could not be localized. All five Cd sites are surrounded by six O atoms, resulting in a distorted octa­hedral environment for each metal atom. The Cd—O bond lengths are in a broad range between 2.184 (6) and 2.599 (6) Å (Table 2 ▸). The mean bond lengths are 2.341 Å (Cd1), 2.283 Å (Cd2), 2.222 Å (Cd3), 2.331 Å (Cd4) and 2.336 Å (Cd5), again in good agreement with the literature value specified above. The BVS values of the Cd atoms amount to 1.91, 2.16, 1.98, 2.00 and 1.90 valence units and thus show good agreement with the expected value of 2.

Of the twelve O sites present in the structure of Cd_5_(PO_4_)_2_(OH)_4_, four are occupied by the O atom of a hydroxide anion, as revealed by BVS calculations. O2, O4, O5, and O8 have considerably lower BVS values of 1.14, 1.17, 1.30 and 1.18 valence units, respectively, than the remaining O atoms [1.78 (O1), 1.96 (O3), 1.97 (O6), 1.90 (O7), 1.94 (O9), 1.95 (O10), 1.71 (O11) and 1.79 (O12) valence units]. Moreover, these four oxygen sites are the only ones that are not part of a phosphate group. Each of the hydroxide O atoms is connected to three Cd atoms in the form of a flat trigonal pyramid. According to the oxygen environments around the hydroxide O atoms, the closest possible acceptor groups for O—H⋯O hydrogen-bonding inter­actions are located at 2.997 (8) Å for O2⋯O1(−*x*, *y* + 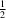 , −*z* + 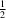 ), 2.986 (9) Å for O4⋯O9(−*x* + 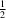 , −*y* + 1, *z* + 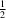 ), 2.890 (9) Å for O5⋯O12(−*x* + 1, *y* + 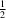 , −*z* + 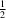 ), and 2.827 (9) Å for O8⋯O11(−*x* + 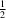 , −*y* + 1, *z* + 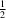 ), indicating rather weak hydrogen bonds in each case.

**Figure 4:**
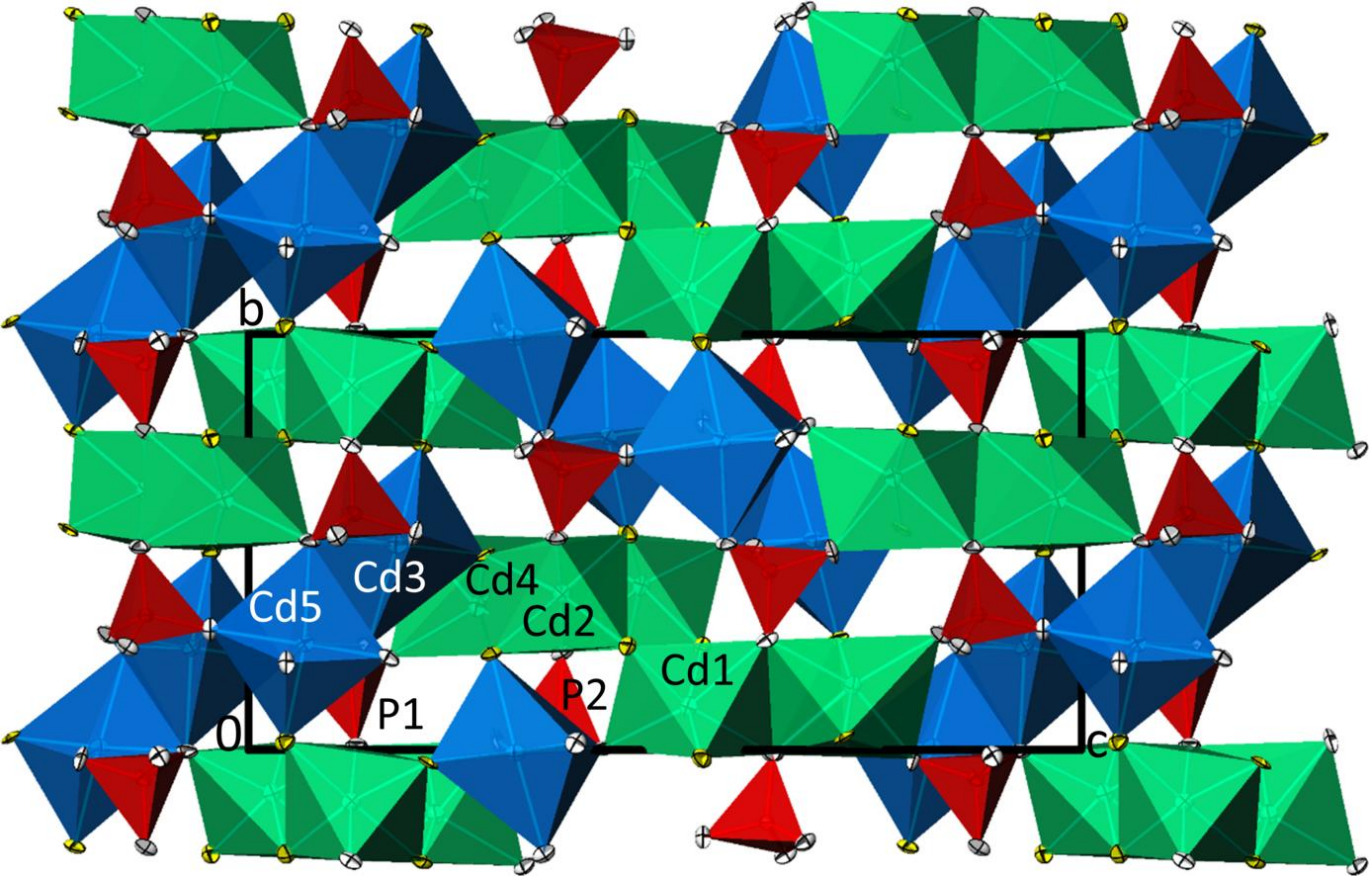
The crystal structure of Cd_5_(PO_4_)_2_(OH)_4_ in a projection along [100] in polyhedral representation. The [CdO_6_] octa­hedra defining the first sub-unit are given in green, the [CdO_6_] octa­hedra defining the second sub-unit are given in blue; [PO_4_] tetra­hedra are red. O atoms of OH groups are yellow, other O atoms are white. Displacement ellipsoids are displayed at the 74% probability level.

The [CdO_6_] polyhedra {[Cd1O_3_(OH)_3_], [Cd2O_2_(OH)_4_], [Cd3O_4_(OH)_2_], [Cd4O_4_(OH)_2_] and [Cd5O_5_(OH)]} define the structure by forming two main sub-units. Through edge-sharing, the octa­hedra around Cd1, Cd2 and Cd4 form corrugated double ribbons extending parallel to [100] as the first unit. The second unit is defined by the octa­hedra around Cd3 and Cd5. By sharing corners and edges, another corrugated ribbon is formed and also propagates parallel to [100]. The two types of ribbons are linked into a tri-periodic arrangement by sharing corners, as well as by sharing the two PO_4_ tetra­hedra (Fig. 4 ▸). The latter show deviations from an ideal tetra­hedral arrangement, as revealed by slightly different bond lengths (Table 2 ▸) and by angular distortions, with O—P—O angles ranging from 106.2 (3) to 112.2 (3)° for P1 and 104.6 (4) to 114.0 (4)° for P2.

Cd_5_(PO_4_)_2_(OH)_4_ and the four isotypic *M*_5_(*X*O_4_)_2_(OH)_4_ crystal structures (*M* = Cd, Mn, Co; *X* = P, As, V) were quanti­tatively compared using the *compstru* software (de la Flor *et al.*, 2016 ▸) available at the Bilbao Crystallographic server (Aroyo *et al.*, 2006 ▸). For this purpose and for direct comparison of bond lengths (Table 2 ▸), the hydrogen atoms (if part of the model) were removed, and all crystal structures were standardized with *STRUCTURE-TIDY* (Gelato & Parthé, 1987 ▸). With Cd_5_(PO_4_)_2_(OH)_4_ as the reference structure, numerical values of parameter of comparison (degree of lattice distortion *S*, the arithmetic mean of the distance between paired atoms *d*_av_, the maximum difference between the atomic positions of the matching atoms *d*_max_, and the measure of similarity *Δ*) are collated in Table 2 ▸. As expected for isotypic structures, the low values for *Δ* indicate high similarities of Cd_5_(PO_4_)_2_(OH)_4_ with the four *M*_5_(*X*O_4_)_2_(OH)_4_ crystal structures. The differences in bond lengths of the individual structural units ([*M*O_6_]; [*X*O_4_]) are due to the different sizes of *M* ^II^ and *X* ^V^, *viz*. 0.745 Å for Co (high spin), 0.83 Å for Mn (high spin), 0.95 Å for Cd, and 0.17 Å for P, 0.335 Å for As, 0.355 Å for V; all values were taken from Shannon (1976 ▸). As a simple measure, the quotient *X*:*M* can be used for correlation. The closer the quotient is to that of P:Cd = 0.178, the higher is the similarity.

## 3. Synthesis and crystallization

Crystals of Cd_2_(PO_4_)OH and Cd_5_(PO_4_)_2_(OH)_4_ were both obtained from reactions under hydro­thermal conditions. The starting materials were 0.1927 g (1.129 mmol) CdCO_3_, 0.1784 g (1.118 mmol) TeO_2_ and 0.1289 g (1.118 mmol) of 85%_wt_ H_3_PO_4_ for the Cd_2_(PO_4_)OH batch, and 0.1874 g (0.607 mmol) Cd(NO_3_)_2_·4H_2_O, 0.0296 g (0.257 mmol) 85%_wt_ H_3_PO_4_, and 0.2197 g (3.916 mmol) KOH for the Cd_5_(PO_4_)_2_(OH)_4_ batch. The reactants were weighed into small Teflon containers with a volume of *ca* 3 ml and mixed with deionized water so that the inner volume was filled to about two thirds with liquid. Then, the Teflon containers were placed into a steel autoclave and heated to 483 K for 7 d. Afterwards the autoclave was cooled down to room temperature within about 4 h. The formed solids were filtered off, washed with mother liquor, water and ethanol, and dried in air.

For the Cd_2_(PO_4_)OH batch, the reaction product was a mixture of a white and bright-yellow solid. An X-ray powder diffraction measurement revealed *α*-TeO_2_, which can be associated with the yellow solid, as a side product besides Cd_2_(PO_4_)OH. Small colourless block-shaped crystals of Cd_2_(PO_4_)OH could be isolated for single crystal X-ray diffraction.

For the Cd_5_(PO_4_)_2_(OH)_4_ batch, the reaction product was a white powder. Apart from Cd(OH)_2_ and Cd_5_(PO_4_)_2_(OH)_4_ no other phases could be identified in the X-ray powder diffraction pattern. Colourless block-shaped crystals of Cd_5_(PO_4_)_2_(OH)_4_ could be isolated for single crystal X-ray diffraction.

## 4. Refinement

Crystal data, data collection and structure refinement details are summarized in Table 3 ▸.

For structure refinement of triplite-type Cd_2_(PO_4_)OH, labelling and fractional coordinates of atoms were adapted from the crystal structure of triplite (Waldrop, 1969 ▸). For direct comparison with other triplite-like structures (Waldrop, 1969 ▸; Đorđević & Kolitsch, 2013 ▸), the unconventional setting *I*2*/a* of space-group type No. 15 was chosen. The conventional setting in *C*2/*c* transforms with −**a** − **c**, −**b**, **c** to the chosen unconventional setting. Oxygen atoms O1 and O2 were found to be positionally disordered over two sites. The pairs O1*A*/O1*B* and O2*A*/O2*B* were refined with common displacement parameters each. The site occupation factors were refined for the pair O1*A*/O2*A* and O1*A*/O2*A* to a ratio of 0.349 (18):0.651 (18). Remaining positive and negative resid­ual electron density close to the Cd1 position suggests possible positional disorder of this atom as well. However, using split positions for Cd1 led to a physically non-meaningful model and was not considered for the final refinement. H atoms could not be located for Cd_2_(PO_4_)OH.

For better comparison with the isotypic crystal structures of *M*_5_(*X*O_4_)_2_(OH)_4_ compounds (*M* = Cd, Mn, Co; *X* = P, As), structure data of Cd_5_(PO_4_)_2_(OH)_4_ were standardized with *STRUCTURE-TIDY* (Gelato & Parthé, 1987 ▸). H atoms could not be located reliably for Cd_5_(PO_4_)_2_(OH)_4_.

## Supplementary Material

Crystal structure: contains datablock(s) CdPO4OH, Cd5PO42OH2, global. DOI: 10.1107/S2056989024000793/tx2081sup1.cif

Structure factors: contains datablock(s) CdPO4OH. DOI: 10.1107/S2056989024000793/tx2081CdPO4OHsup2.hkl

Structure factors: contains datablock(s) Cd5PO42OH2. DOI: 10.1107/S2056989024000793/tx2081Cd5PO42OH2sup3.hkl

CCDC references: 2327315, 2327314

Additional supporting information:  crystallographic information; 3D view; checkCIF report

## Figures and Tables

**Table 1:** Selected bond lengths (Å) for Cd_2_(PO_4_)OH

Cd1—O3^i^	2.224 (7)	Cd2—O1*B* ^iv^	2.242 (12)
Cd1—OH	2.256 (6)	Cd2—O4	2.251 (7)
Cd1—O4^ii^	2.275 (6)	Cd2—O2*B* ^iii^	2.38 (5)
Cd1—O2*B* ^iii^	2.28 (5)	Cd2—O1*B* ^vi^	2.556 (14)
Cd1—O3^iv^	2.350 (6)	P1—O2*B* ^vii^	1.51 (5)
Cd1—OH^i^	2.484 (7)	P1—O3	1.519 (7)
Cd2—OH^v^	2.101 (6)	P1—O1*B*	1.528 (11)
Cd2—OH	2.174 (7)	P1—O4	1.542 (7)

Symmetry codes: (i) 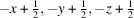 ; (ii) 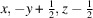 ; (iii) 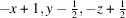 ; (iv) 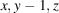 ; (v) 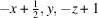 ; (vi) 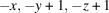 ; (vii) 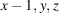 .

**Table 2:** Comparison of bond lengths (Å) in the isotypic *M*_5_(*X*O_4_)_2_(OH)_4_ structures (*M* = Cd, Mn, Co; *X* = P, As, V) after standardization, and parameters of structural comparison with Cd_5_(PO_4_)_2_(OH)_4_ as the reference structure

	Cd_5_(PO_4_)_2_(OH)_4_	Mn_5_(AsO_4_)_2_(OH)_4_^*a*^	Mn_5_(PO_4_)_2_(OH)_4_^*b*^	Co_5_(PO_4_)_2_(OH)_4_^*c*^	Cd_5_(VO_4_)_2_(OH)_4_^*d*^
*M*1—O8	2.245 (7)	2.19	2.156	2.063	2.295 (4)
*M*1—O4^i^	2.267 (6)	2.25	2.193	2.132	2.317 (4)
*M*1—O8^i^	2.269 (6)	2.17	2.157	2.050	2.271 (4)
*M*1—O3	2.312 (6)	2.20	2.214	2.207	2.287 (3)
*M*1—O6^ii^	2.412 (7)	2.31	2.299	2.162	2.378 (4)
*M*1—O10^i^	2.540 (6)	2.35	2.407	2.250	2.408 (3)
*M*2—O8^i^	2.236 (6)	2.19	2.153	2.106	2.244 (4)
*M*2—O4	2.246 (6)	2.13	2.131	2.047	2.2469 (4)
*M*2—O5	2.258 (6)	2.19	2.166	2.107	2.265 (4)
*M*2—O2^iii^	2.306 (6)	2.18	2.181	2.061	2.293 (4)
*M*2—O6^iv^	2.319 (6)	2.19	2.229	2.171	2.321 (3)
*M*2—O10	2.335 (6)	2.25	2.244	2.152	2.329 (3)
*M*3—O5	2.202 (6)	2.07	2.076	1.998	2.193 (3)
*M*3—O11	2.226 (6)	2.06	2.076	1.983	2.212 (3)
*M*3—O2	2.300 (6)	2.26	2.225	2.232	2.302 (4)
*M*3—O7^iv^	2.327 (6)	2.13	2.179	2.085	2.268 (4)
*M*3—O1	2.351 (6)	2.31	2.293	2.194	2.325 (3)
*M*3—O3^iv^	2.599 (6)	2.69	2.583	2.410	2.849 (4)
*M*4—O5	2.184 (6)	2.15	2.107	2.029	2.241 (4)
*M*4—O2^v^	2.196 (6)	2.14	2.116	2.031	2.252 (3)
*M*4—O7^vi^	2.305 (6)	2.18	2.173	2.078	2.282 (3)
*M*4—O12	2.315 (6)	2.22	2.210	2.170	2.302 (3)
*M*4—O10	2.434 (6)	2.29	2.328	2.194	2.365 (4)
*M*4—O6^vi^	2.555 (6)	2.36	2.453	2.340	2.413 (4)
*M*5—O4^v^	2.240 (7)	2.17	2.165	2.110	2.245 (4)
*M*5—O9^vii^	2.247 (6)	2.09	2.098	2.029	2.256 (4)
*M*5—O3^vi^	2.334 (6)	2.32	2.297	2.260	2.299 (4)
*M*5—O1^viii^	2.364 (6)	2.19	2.233	2.092	2.287 (3)
*M*5—O9	2.405 (6)	2.19	2.259	2.114	2.381 (4)
*M*5—O12	2.432 (6)	2.43	2.384	2.281	2.642 (4)
*X*1—O9	1.524 (6)	1.72	1.547	1.537	1.698 (3)
*X*1—O6	1.543 (7)	1.72	1.554	1.547	1.741 (4)
*X*1—O12	1.544 (6)	1.65	1.528	1.545	1.695 (4)
*X*1—O1	1.553 (6)	1.68	1.539	1.552	1.749 (3)
*X*2—O11^v^	1.523 (6)	1.65	1.527	1.519	1.688 (3)
*X*2—O7	1.538 (7)	1.67	1.540	1.546	1.721 (3)
*X*2—O10	1.543 (7)	1.67	1.544	1.545	1.731 (4)
*X*2—O3	1.562 (6)	1.68	1.542	1.555	1.733 (4)
					
*S*		0.0118	0.0199	0.0394	0.0106
*d*_max_		0.3033	0.1232	0.2613	0.2351
*d*_av_		0.1378	0.0598	0.1123	0.1264
*Δ*		0.044	0.013	0.026	0.100
quotient *X*:*M* of ionic radii	0.178	0.404	0.205	0.228	0.374

Notes: (*a*) Lattice parameter after standardization: *a* = 5.75 (1), *b* = 9.31 (2), *c* = 18.29 (2) Å, *V* = 979.1 Å^3^. (*b*) Lattice parameters after standardization: *a* = 5.6923 (6), *b* = 9.110 (1), *c* = 18.032 (4) Å, *V* = 935.1 Å^3^. (*c*) Lattice parameters after standardization: *a* = 5.5154 (4), *b* = 8.903 (2), *c* = 17.397 (2) Å, *V* = 854.3 Å^3^. (*d*) Lattice parameters after standardization: *a* = 6.0133 (12), *b* = 9.5411 (19) Å, *c* = 19.011 (4) Å, *V* = 1090.7 (4) Å^3^. Symmetry codes: (i) *x* − 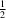 , −*y* + 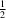 , −*z* + 1; (ii) −*x* + 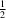 , −*y*, *z* + 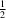 ; (iii) −*x*, *y* − 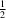 , −*z* + 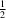 ; (iv) −*x*, *y* + 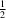 , −*z* + 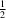 ; (v) −*x* + 1, *y* − 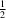 , −*z* + 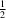 ; (vi) −*x* + 1, *y* + 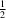 , −*z* + 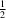 ; (vii) *x* + 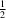 , −*y* + 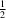 , −*z*; (viii) *x* + 1, *y*, *z*.

**Table 3:** Experimental details

	Cd_2_(PO_4_)OH	Cd_5_(PO_4_)_2_(OH)_4_
Crystal data		
*M*_r_	336.78	819.97
Crystal system, space group	Monoclinic, *I*2/*a*	Orthorhombic, *P*2_1_2_1_2_1_
Temperature (K)	296	296
*a*, *b*, *c* (Å)	12.4307 (13), 6.6910 (6), 10.7087 (10)	5.8901 (4), 9.3455 (6), 18.7423 (13)
α, β, γ (°)	90, 107.506 (3), 90	90, 90, 90
*V* (Å^3^)	849.43 (14)	1031.69 (12)
*Z*	8	4
Radiation type	Mo *K*α	Mo *K*α
μ (mm^−1^)	10.30	10.51
Crystal size (mm)	0.10 × 0.08 × 0.05	0.11 × 0.07 × 0.04

Data collection		
Diffractometer	Bruker APEXII CCD	Bruker APEXII CCD
Absorption correction	Multi-scan (*SADABS*; Krause *et al.*, 2015 ▸)	Multi-scan (*SADABS*; Krause *et al.*, 2015 ▸)
*T*_min_, *T*_max_	0.600, 0.746	0.517, 0.747
No. of measured, independent and observed [*I* > 2σ(*I*)] reflections	7833, 1477, 1026	10057, 4493, 3588
*R*_int_	0.065	0.051
(sin θ/λ)_max_ (Å^−1^)	0.747	0.806

Refinement		
*R*[*F* ^2^ > 2σ(*F* ^2^)], *wR*(*F* ^2^), *S*	0.047, 0.100, 1.03	0.039, 0.059, 0.97
No. of reflections	1477	4493
No. of parameters	80	172
H-atom treatment	H-atom parameters not defined	H-atom parameters not defined
Δρ_max_, Δρ_min_ (e Å^−3^)	2.91, −3.55	1.53, −1.76
Absolute structure	–	Flack *x* determined using 1284 quotients [(*I* ^+^)−(*I* ^−^)]/[(*I* ^+^)+(*I* ^−^)] (Parsons *et al.*, 2013 ▸)
Absolute structure parameter	–	−0.03 (4)

Computer programs: *APEX3* and *SAINT* (Bruker, 2016 ▸), SHELXT (Sheldrick, 2015*a* ▸), *SHELXL* (Sheldrick, 2015*b* ▸), *ATOMS* (Dowty, 2006 ▸) and *publCIF* (Westrip, 2010 ▸).

## supplementary crystallographic information

### Dicadmium orthophosphate hydroxide (CdPO4OH) Crystal data

**Table:**

Cd_2_(PO_4_)OH	*F*(000) = 1216
*M**_r_* = 336.78	*D*_x_ = 5.267 Mg m^−^^3^
Monoclinic, *I*2/*a*	Mo *K*α radiation, λ = 0.71073 Å
*a* = 12.4307 (13) Å	Cell parameters from 1272 reflections
*b* = 6.6910 (6) Å	θ = 3.4–25.8°
*c* = 10.7087 (10) Å	µ = 10.30 mm^−^^1^
β = 107.506 (3)°	*T* = 296 K
*V* = 849.43 (14) Å^3^	Block, colourless
*Z* = 8	0.10 × 0.08 × 0.05 mm

### Dicadmium orthophosphate hydroxide (CdPO4OH) Data collection

**Table:**

Bruker APEXII CCD diffractometer	1026 reflections with *I* > 2σ(*I*)
ω– and φ–scan	*R*_int_ = 0.065
Absorption correction: multi-scan (SADABS; Krause *et al.*, 2015)	θ_max_ = 32.1°, θ_min_ = 3.4°
*T*_min_ = 0.600, *T*_max_ = 0.746	*h* = −18→18
7833 measured reflections	*k* = −9→9
1477 independent reflections	*l* = −15→15

### Dicadmium orthophosphate hydroxide (CdPO4OH) Refinement

**Table:**

Refinement on *F*^2^	80 parameters
Least-squares matrix: full	0 restraints
*R*[*F*^2^ > 2σ(*F*^2^)] = 0.047	*w* = 1/[σ^2^(*F*_o_^2^) + (0.0267*P*)^2^ + 36.2119*P*] where *P* = (*F*_o_^2^ + 2*F*_c_^2^)/3
*wR*(*F*^2^) = 0.100	(Δ/σ)_max_ < 0.001
*S* = 1.03	Δρ_max_ = 2.91 e Å^−^^3^
1477 reflections	Δρ_min_ = −3.55 e Å^−^^3^

### Dicadmium orthophosphate hydroxide (CdPO4OH) Special details

**Table:**

Geometry. All esds (except the esd in the dihedral angle between two l.s. planes) are estimated using the full covariance matrix. The cell esds are taken into account individually in the estimation of esds in distances, angles and torsion angles; correlations between esds in cell parameters are only used when they are defined by crystal symmetry. An approximate (isotropic) treatment of cell esds is used for estimating esds involving l.s. planes.

### Dicadmium orthophosphate hydroxide (CdPO4OH) Fractional atomic coordinates and isotropic or equivalent isotropic displacement parameters (Å^2^)

**Table:**

	*x*	*y*	*z*	*U*_iso_*/*U*_eq_	Occ. (<1)
Cd1	0.20094 (5)	−0.00573 (10)	0.19098 (6)	0.01905 (15)	
Cd2	0.10811 (8)	0.15931 (10)	0.44546 (7)	0.0293 (2)	
P1	0.07732 (19)	0.6694 (3)	0.3759 (2)	0.0151 (4)	
O1A	0.123 (2)	0.856 (3)	0.469 (2)	0.028 (3)	0.349 (18)
O1B	0.0590 (13)	0.8402 (17)	0.4621 (11)	0.028 (3)	0.651 (18)
O2A	0.970 (8)	0.592 (15)	0.297 (10)	0.040 (7)	0.349 (18)
O2B	0.958 (4)	0.640 (7)	0.287 (5)	0.040 (7)	0.651 (18)
O3	0.1538 (6)	0.7093 (10)	0.2916 (7)	0.0299 (16)	
O4	0.1231 (6)	0.4933 (10)	0.4698 (6)	0.0261 (14)	
OH	0.2574 (6)	0.1685 (10)	0.3799 (6)	0.0265 (14)	

### Dicadmium orthophosphate hydroxide (CdPO4OH) Atomic displacement parameters (Å^2^)

**Table:**

	*U*^11^	*U*^22^	*U*^33^	*U*^12^	*U*^13^	*U*^23^
Cd1	0.0173 (3)	0.0217 (3)	0.0184 (3)	0.0027 (3)	0.0058 (2)	0.0029 (3)
Cd2	0.0639 (6)	0.0122 (3)	0.0167 (3)	0.0014 (3)	0.0197 (3)	−0.0001 (3)
P1	0.0196 (11)	0.0113 (8)	0.0157 (9)	0.0003 (8)	0.0072 (8)	−0.0014 (8)
O1A	0.043 (8)	0.017 (4)	0.025 (4)	0.009 (6)	0.012 (6)	−0.004 (3)
O1B	0.043 (8)	0.017 (4)	0.025 (4)	0.009 (6)	0.012 (6)	−0.004 (3)
O2A	0.011 (10)	0.08 (2)	0.029 (9)	0.002 (11)	0.000 (8)	0.033 (12)
O2B	0.011 (10)	0.08 (2)	0.029 (9)	0.002 (11)	0.000 (8)	0.033 (12)
O3	0.019 (4)	0.030 (4)	0.046 (4)	0.002 (3)	0.019 (3)	0.014 (3)
O4	0.041 (4)	0.018 (3)	0.020 (3)	0.007 (3)	0.011 (3)	0.005 (3)
OH	0.040 (4)	0.023 (3)	0.014 (3)	0.009 (3)	0.003 (3)	−0.003 (3)

### Dicadmium orthophosphate hydroxide (CdPO4OH) Geometric parameters (Å, º)

**Table:**

Cd1—O3^i^	2.224 (7)	Cd2—O4	2.251 (7)
Cd1—OH	2.256 (6)	Cd2—O2B^ii^	2.38 (5)
Cd1—O2A^ii^	2.26 (10)	Cd2—O2A^ii^	2.52 (9)
Cd1—O4^iii^	2.275 (6)	Cd2—O1B^vi^	2.556 (14)
Cd1—O2B^ii^	2.28 (5)	Cd2—Cd2^v^	3.3659 (18)
Cd1—O3^iv^	2.350 (6)	P1—O2A^vii^	1.44 (11)
Cd1—OH^i^	2.484 (7)	P1—O2B^vii^	1.51 (5)
Cd1—Cd2^iii^	3.4339 (9)	P1—O3	1.519 (7)
Cd1—Cd2	3.4453 (10)	P1—O1B	1.528 (11)
Cd2—O1A^iv^	2.05 (2)	P1—O4	1.542 (7)
Cd2—OH^v^	2.101 (6)	P1—O1A	1.59 (2)
Cd2—OH	2.174 (7)	O1A—O1B	0.78 (2)
Cd2—O1B^iv^	2.242 (12)		
			
O3^i^—Cd1—OH	102.4 (3)	O1B^iv^—Cd2—Cd2^v^	104.7 (4)
O3^i^—Cd1—O2A^ii^	158 (3)	O4—Cd2—Cd2^v^	85.30 (18)
OH—Cd1—O2A^ii^	81.1 (17)	O2B^ii^—Cd2—Cd2^v^	111.2 (12)
O3^i^—Cd1—O4^iii^	100.9 (3)	O2A^ii^—Cd2—Cd2^v^	113 (2)
OH—Cd1—O4^iii^	146.6 (2)	O1B^vi^—Cd2—Cd2^v^	139.0 (3)
O2A^ii^—Cd1—O4^iii^	86 (3)	O1A^iv^—Cd2—Cd1^viii^	125.3 (6)
O3^i^—Cd1—O2B^ii^	163.9 (15)	OH^v^—Cd2—Cd1^viii^	45.93 (19)
OH—Cd1—O2B^ii^	74.4 (8)	OH—Cd2—Cd1^viii^	96.04 (17)
O2A^ii^—Cd1—O2B^ii^	9 (2)	O1B^iv^—Cd2—Cd1^viii^	128.2 (3)
O4^iii^—Cd1—O2B^ii^	89.0 (13)	O4—Cd2—Cd1^viii^	40.91 (16)
O3^i^—Cd1—O3^iv^	76.9 (2)	O2B^ii^—Cd2—Cd1^viii^	140.2 (11)
OH—Cd1—O3^iv^	93.5 (3)	O2A^ii^—Cd2—Cd1^viii^	148 (2)
O2A^ii^—Cd1—O3^iv^	81 (3)	O1B^vi^—Cd2—Cd1^viii^	80.1 (3)
O4^iii^—Cd1—O3^iv^	114.9 (3)	Cd2^v^—Cd2—Cd1^viii^	70.04 (2)
O2B^ii^—Cd1—O3^iv^	87.5 (14)	O1A^iv^—Cd2—Cd1	75.0 (7)
O3^i^—Cd1—OH^i^	110.1 (2)	OH^v^—Cd2—Cd1	110.3 (2)
OH—Cd1—OH^i^	76.5 (2)	OH—Cd2—Cd1	39.81 (17)
O2A^ii^—Cd1—OH^i^	92 (3)	O1B^iv^—Cd2—Cd1	85.5 (3)
O4^iii^—Cd1—OH^i^	73.2 (2)	O4—Cd2—Cd1	111.95 (16)
O2B^ii^—Cd1—OH^i^	84.8 (13)	O2B^ii^—Cd2—Cd1	41.3 (12)
O3^iv^—Cd1—OH^i^	168.8 (2)	O2A^ii^—Cd2—Cd1	41 (2)
O3^i^—Cd1—Cd2^iii^	123.70 (17)	O1B^vi^—Cd2—Cd1	144.2 (3)
OH—Cd1—Cd2^iii^	106.25 (17)	Cd2^v^—Cd2—Cd1	73.08 (3)
O2A^ii^—Cd1—Cd2^iii^	75 (3)	Cd1^viii^—Cd2—Cd1	134.96 (3)
O4^iii^—Cd1—Cd2^iii^	40.40 (16)	O2A^vii^—P1—O2B^vii^	14 (4)
O2B^ii^—Cd1—Cd2^iii^	71.9 (16)	O2A^vii^—P1—O3	110 (4)
O3^iv^—Cd1—Cd2^iii^	145.72 (17)	O2B^vii^—P1—O3	108 (2)
OH^i^—Cd1—Cd2^iii^	37.43 (15)	O2A^vii^—P1—O1B	110 (5)
O3^i^—Cd1—Cd2	125.92 (19)	O2B^vii^—P1—O1B	101 (2)
OH—Cd1—Cd2	38.11 (19)	O3—P1—O1B	117.5 (6)
O2A^ii^—Cd1—Cd2	47 (2)	O2A^vii^—P1—O4	102 (3)
O4^iii^—Cd1—Cd2	132.32 (18)	O2B^vii^—P1—O4	114.4 (14)
O2B^ii^—Cd1—Cd2	43.4 (12)	O3—P1—O4	110.3 (4)
O3^iv^—Cd1—Cd2	73.04 (18)	O1B—P1—O4	105.7 (5)
OH^i^—Cd1—Cd2	95.77 (14)	O2A^vii^—P1—O1A	138 (4)
Cd2^iii^—Cd1—Cd2	105.90 (2)	O2B^vii^—P1—O1A	126 (2)
O1A^iv^—Cd2—OH^v^	84.3 (7)	O3—P1—O1A	93.8 (10)
O1A^iv^—Cd2—OH	90.8 (8)	O1B—P1—O1A	29.0 (8)
OH^v^—Cd2—OH	76.1 (3)	O4—P1—O1A	101.5 (9)
O1A^iv^—Cd2—O1B^iv^	20.4 (7)	O1B—O1A—P1	71 (2)
OH^v^—Cd2—O1B^iv^	96.9 (4)	O1B—O1A—Cd2^ix^	94 (2)
OH—Cd2—O1B^iv^	109.4 (4)	P1—O1A—Cd2^ix^	134.3 (14)
O1A^iv^—Cd2—O4	165.7 (7)	O1A—O1B—P1	80 (2)
OH^v^—Cd2—O4	81.5 (2)	O1A—O1B—Cd2^ix^	66 (2)
OH—Cd2—O4	87.7 (2)	P1—O1B—Cd2^ix^	124.8 (7)
O1B^iv^—Cd2—O4	162.0 (4)	O1A—O1B—Cd2^vi^	152 (2)
O1A^iv^—Cd2—O2B^ii^	93.8 (13)	P1—O1B—Cd2^vi^	121.4 (8)
OH^v^—Cd2—O2B^ii^	150.0 (12)	Cd2^ix^—O1B—Cd2^vi^	107.6 (4)
OH—Cd2—O2B^ii^	74.0 (12)	P1^x^—O2A—Cd1^xi^	142 (7)
O1B^iv^—Cd2—O2B^ii^	90.9 (11)	P1^x^—O2A—Cd2^xi^	123 (5)
O4—Cd2—O2B^ii^	99.5 (12)	Cd1^xi^—O2A—Cd2^xi^	92 (3)
O1A^iv^—Cd2—O2A^ii^	87 (3)	P1^x^—O2B—Cd1^xi^	135 (2)
OH^v^—Cd2—O2A^ii^	151 (2)	P1^x^—O2B—Cd2^xi^	128 (3)
OH—Cd2—O2A^ii^	77 (2)	Cd1^xi^—O2B—Cd2^xi^	95.3 (19)
O1B^iv^—Cd2—O2A^ii^	83 (2)	P1—O3—Cd1^i^	118.7 (4)
O4—Cd2—O2A^ii^	107 (2)	P1—O3—Cd1^ix^	134.6 (4)
O2B^ii^—Cd2—O2A^ii^	8 (3)	Cd1^i^—O3—Cd1^ix^	103.1 (2)
O1A^iv^—Cd2—O1B^vi^	90.4 (8)	P1—O4—Cd2	133.0 (4)
OH^v^—Cd2—O1B^vi^	100.2 (3)	P1—O4—Cd1^viii^	127.5 (4)
OH—Cd2—O1B^vi^	175.9 (3)	Cd2—O4—Cd1^viii^	98.7 (2)
O1B^iv^—Cd2—O1B^vi^	72.4 (4)	Cd2^v^—OH—Cd2	103.8 (3)
O4—Cd2—O1B^vi^	90.2 (3)	Cd2^v^—OH—Cd1	137.1 (3)
O2B^ii^—Cd2—O1B^vi^	109.8 (12)	Cd2—OH—Cd1	102.1 (3)
O2A^ii^—Cd2—O1B^vi^	107 (2)	Cd2^v^—OH—Cd1^i^	96.6 (3)
O1A^iv^—Cd2—Cd2^v^	84.9 (8)	Cd2—OH—Cd1^i^	113.6 (3)
OH^v^—Cd2—Cd2^v^	38.84 (19)	Cd1—OH—Cd1^i^	103.5 (2)
OH—Cd2—Cd2^v^	37.31 (16)		

Symmetry codes: (i) −*x*+1/2, −*y*+1/2, −*z*+1/2; (ii) −*x*+1, *y*−1/2, −*z*+1/2; (iii) *x*, −*y*+1/2, *z*−1/2; (iv) *x*, *y*−1, *z*; (v) −*x*+1/2, *y*, −*z*+1; (vi) −*x*, −*y*+1, −*z*+1; (vii) *x*−1, *y*, *z*; (viii) *x*, −*y*+1/2, *z*+1/2; (ix) *x*, *y*+1, *z*; (x) *x*+1, *y*, *z*; (xi) −*x*+1, *y*+1/2, −*z*+1/2.

### Pentacadmium bis(orthophosphate) tetrakis(hydroxide) (Cd5PO42OH2) Crystal data

**Table:**

Cd_5_(PO_4_)_2_(OH)_4_	*D*_x_ = 5.279 Mg m^−^^3^
*M**_r_* = 819.97	Mo *K*α radiation, λ = 0.71073 Å
Orthorhombic, *P*2_1_2_1_2_1_	Cell parameters from 2236 reflections
*a* = 5.8901 (4) Å	θ = 3.9–33.6°
*b* = 9.3455 (6) Å	µ = 10.51 mm^−^^1^
*c* = 18.7423 (13) Å	*T* = 296 K
*V* = 1031.69 (12) Å^3^	Block, colourless
*Z* = 4	0.11 × 0.07 × 0.04 mm
*F*(000) = 1480	

### Pentacadmium bis(orthophosphate) tetrakis(hydroxide) (Cd5PO42OH2) Data collection

**Table:**

Bruker APEXII CCD diffractometer	3588 reflections with *I* > 2σ(*I*)
ω– and φ–scan	*R*_int_ = 0.051
Absorption correction: multi-scan (SADABS; Krause *et al.*, 2015)	θ_max_ = 35.0°, θ_min_ = 2.2°
*T*_min_ = 0.517, *T*_max_ = 0.747	*h* = −9→9
10057 measured reflections	*k* = −14→11
4493 independent reflections	*l* = −28→30

### Pentacadmium bis(orthophosphate) tetrakis(hydroxide) (Cd5PO42OH2) Refinement

**Table:**

Refinement on *F*^2^	H-atom parameters not defined
Least-squares matrix: full	*w* = 1/[σ^2^(*F*_o_^2^) + (0.0115*P*)^2^] where *P* = (*F*_o_^2^ + 2*F*_c_^2^)/3
*R*[*F*^2^ > 2σ(*F*^2^)] = 0.039	(Δ/σ)_max_ < 0.001
*wR*(*F*^2^) = 0.059	Δρ_max_ = 1.53 e Å^−^^3^
*S* = 0.97	Δρ_min_ = −1.76 e Å^−^^3^
4493 reflections	Absolute structure: Flack *x* determined using 1284 quotients [(*I*^+^)-(*I*^-^)]/[(*I*^+^)+(*I*^-^)] (Parsons *et al.*, 2013)
172 parameters	Absolute structure parameter: −0.03 (4)
0 restraints	

### Pentacadmium bis(orthophosphate) tetrakis(hydroxide) (Cd5PO42OH2) Special details

**Table:**

Geometry. All esds (except the esd in the dihedral angle between two l.s. planes) are estimated using the full covariance matrix. The cell esds are taken into account individually in the estimation of esds in distances, angles and torsion angles; correlations between esds in cell parameters are only used when they are defined by crystal symmetry. An approximate (isotropic) treatment of cell esds is used for estimating esds involving l.s. planes.

### Pentacadmium bis(orthophosphate) tetrakis(hydroxide) (Cd5PO42OH2) Fractional atomic coordinates and isotropic or equivalent isotropic displacement parameters (Å^2^)

**Table:**

	*x*	*y*	*z*	*U*_iso_*/*U*_eq_	
Cd1	0.05007 (11)	0.10420 (7)	0.52815 (3)	0.01113 (12)	
Cd2	0.05248 (11)	0.37573 (7)	0.37637 (4)	0.01083 (12)	
Cd3	0.08667 (9)	0.49327 (7)	0.17892 (4)	0.01161 (13)	
Cd4	0.56114 (11)	0.34489 (7)	0.27458 (3)	0.01154 (12)	
Cd5	0.76352 (10)	0.25492 (7)	0.06812 (4)	0.01085 (12)	
P1	0.3047 (3)	0.1745 (2)	0.12271 (13)	0.0081 (4)	
P2	0.3795 (3)	0.0690 (2)	0.37363 (13)	0.0084 (4)	
O1	0.0817 (9)	0.2510 (7)	0.1450 (3)	0.0120 (12)	
O2	0.1169 (10)	0.7316 (6)	0.2081 (3)	0.0111 (12)	
O3	0.2013 (10)	0.0034 (7)	0.4260 (3)	0.0130 (12)	
O4	0.2237 (11)	0.5202 (7)	0.4556 (3)	0.0126 (13)	
O5	0.2489 (11)	0.4695 (6)	0.2841 (3)	0.0117 (12)	
O6	0.2772 (10)	0.0109 (7)	0.1297 (4)	0.0126 (12)	
O7	0.2995 (10)	0.0217 (7)	0.2992 (3)	0.0146 (13)	
O8	0.3482 (10)	0.2500 (7)	0.5450 (3)	0.0130 (12)	
O9	0.3680 (11)	0.2095 (7)	0.0458 (3)	0.0128 (13)	
O10	0.3790 (9)	0.2340 (7)	0.3772 (3)	0.0113 (12)	
O11	0.3843 (10)	0.5159 (7)	0.1066 (3)	0.0134 (13)	
O12	0.5088 (9)	0.2213 (7)	0.1688 (3)	0.0115 (13)	

### Pentacadmium bis(orthophosphate) tetrakis(hydroxide) (Cd5PO42OH2) Atomic displacement parameters (Å^2^)

**Table:**

	*U*^11^	*U*^22^	*U*^33^	*U*^12^	*U*^13^	*U*^23^
Cd1	0.0100 (2)	0.0129 (3)	0.0104 (3)	−0.0005 (2)	−0.0002 (2)	0.0010 (2)
Cd2	0.0097 (2)	0.0114 (3)	0.0114 (3)	−0.0002 (2)	0.0002 (2)	−0.0006 (2)
Cd3	0.0107 (2)	0.0103 (3)	0.0139 (3)	−0.0001 (2)	−0.0008 (2)	−0.0001 (3)
Cd4	0.0108 (2)	0.0135 (3)	0.0103 (3)	0.0018 (2)	0.0004 (2)	0.0008 (2)
Cd5	0.0124 (2)	0.0096 (3)	0.0105 (3)	−0.0012 (2)	0.0008 (2)	0.0005 (3)
P1	0.0085 (8)	0.0067 (10)	0.0090 (10)	−0.0001 (7)	−0.0006 (7)	0.0002 (9)
P2	0.0078 (8)	0.0085 (10)	0.0089 (10)	0.0015 (7)	0.0008 (7)	0.0013 (9)
O1	0.008 (2)	0.012 (3)	0.016 (3)	0.002 (3)	0.000 (2)	−0.004 (3)
O2	0.011 (2)	0.007 (3)	0.015 (3)	−0.003 (2)	0.002 (2)	−0.003 (3)
O3	0.015 (3)	0.007 (3)	0.016 (3)	−0.003 (2)	0.006 (2)	0.001 (3)
O4	0.013 (3)	0.012 (3)	0.013 (3)	0.000 (2)	−0.003 (2)	−0.002 (3)
O5	0.016 (3)	0.006 (3)	0.013 (3)	−0.001 (2)	0.001 (2)	0.006 (2)
O6	0.016 (3)	0.007 (3)	0.016 (3)	−0.003 (3)	−0.004 (2)	0.000 (3)
O7	0.017 (3)	0.020 (4)	0.007 (3)	0.004 (3)	−0.001 (2)	−0.005 (3)
O8	0.016 (3)	0.012 (3)	0.011 (3)	0.001 (3)	0.002 (2)	0.001 (3)
O9	0.013 (3)	0.017 (4)	0.008 (3)	0.001 (2)	0.000 (2)	0.000 (3)
O10	0.007 (2)	0.010 (3)	0.016 (3)	−0.004 (2)	−0.001 (2)	0.004 (3)
O11	0.011 (2)	0.015 (3)	0.015 (3)	−0.003 (2)	0.001 (2)	−0.001 (3)
O12	0.010 (3)	0.011 (3)	0.014 (3)	0.000 (2)	−0.003 (2)	−0.004 (3)

### Pentacadmium bis(orthophosphate) tetrakis(hydroxide) (Cd5PO42OH2) Geometric parameters (Å, º)

**Table:**

Cd1—O8	2.245 (7)	Cd4—O2^v^	2.196 (6)
Cd1—O4^i^	2.267 (6)	Cd4—O7^vi^	2.305 (6)
Cd1—O8^i^	2.269 (6)	Cd4—O12	2.315 (6)
Cd1—O3	2.312 (6)	Cd4—O10	2.434 (6)
Cd1—O6^ii^	2.412 (7)	Cd4—O6^vi^	2.555 (6)
Cd1—O10^i^	2.540 (6)	Cd5—O4^v^	2.240 (7)
Cd2—O8^i^	2.236 (6)	Cd5—O9^vii^	2.247 (6)
Cd2—O4	2.246 (6)	Cd5—O3^vi^	2.334 (6)
Cd2—O5	2.258 (6)	Cd5—O1^viii^	2.364 (6)
Cd2—O2^iii^	2.306 (6)	Cd5—O9	2.405 (6)
Cd2—O6^iv^	2.319 (6)	Cd5—O12	2.432 (6)
Cd2—O10	2.335 (6)	P1—O9	1.524 (6)
Cd3—O5	2.202 (6)	P1—O6	1.543 (7)
Cd3—O11	2.226 (6)	P1—O12	1.544 (6)
Cd3—O2	2.300 (6)	P1—O1	1.553 (6)
Cd3—O7^iv^	2.327 (6)	P2—O11^v^	1.523 (6)
Cd3—O1	2.351 (6)	P2—O7	1.538 (7)
Cd3—O3^iv^	2.599 (6)	P2—O10	1.543 (7)
Cd4—O5	2.184 (6)	P2—O3	1.562 (6)
			
O8—Cd1—O4^i^	162.9 (2)	O3^vi^—Cd5—O1^viii^	85.2 (2)
O8—Cd1—O8^i^	97.50 (11)	O4^v^—Cd5—O9	79.9 (2)
O4^i^—Cd1—O8^i^	86.8 (2)	O9^vii^—Cd5—O9	97.25 (17)
O8—Cd1—O3	93.6 (2)	O3^vi^—Cd5—O9	105.6 (2)
O4^i^—Cd1—O3	103.2 (2)	O1^viii^—Cd5—O9	150.6 (2)
O8^i^—Cd1—O3	86.9 (2)	O4^v^—Cd5—O12	92.8 (2)
O8—Cd1—O6^ii^	80.2 (2)	O9^vii^—Cd5—O12	157.8 (2)
O4^i^—Cd1—O6^ii^	91.3 (2)	O3^vi^—Cd5—O12	98.4 (2)
O8^i^—Cd1—O6^ii^	165.0 (2)	O1^viii^—Cd5—O12	90.79 (19)
O3—Cd1—O6^ii^	108.0 (2)	O9—Cd5—O12	60.93 (19)
O8—Cd1—O10^i^	81.4 (2)	O9—P1—O6	108.6 (4)
O4^i^—Cd1—O10^i^	82.8 (2)	O9—P1—O12	106.2 (3)
O8^i^—Cd1—O10^i^	81.8 (2)	O6—P1—O12	108.3 (3)
O3—Cd1—O10^i^	166.9 (2)	O9—P1—O1	111.3 (4)
O6^ii^—Cd1—O10^i^	83.2 (2)	O6—P1—O1	110.2 (3)
O8^i^—Cd2—O4	97.0 (2)	O12—P1—O1	112.2 (3)
O8^i^—Cd2—O5	169.9 (2)	O11^v^—P2—O7	114.0 (4)
O4—Cd2—O5	92.5 (2)	O11^v^—P2—O10	108.5 (3)
O8^i^—Cd2—O2^iii^	85.0 (2)	O7—P2—O10	109.0 (4)
O4—Cd2—O2^iii^	178.0 (2)	O11^v^—P2—O3	109.5 (4)
O5—Cd2—O2^iii^	85.6 (2)	O7—P2—O3	104.6 (4)
O8^i^—Cd2—O6^iv^	82.4 (2)	O10—P2—O3	111.3 (4)
O4—Cd2—O6^iv^	94.7 (2)	P1—O1—Cd3	120.4 (3)
O5—Cd2—O6^iv^	100.3 (2)	P1—O1—Cd5^ix^	120.9 (3)
O2^iii^—Cd2—O6^iv^	85.5 (2)	Cd3—O1—Cd5^ix^	99.2 (2)
O8^i^—Cd2—O10	98.1 (2)	Cd4^vi^—O2—Cd3	124.7 (3)
O4—Cd2—O10	88.1 (2)	Cd4^vi^—O2—Cd2^iv^	101.1 (2)
O5—Cd2—O10	78.7 (2)	Cd3—O2—Cd2^iv^	111.7 (2)
O2^iii^—Cd2—O10	91.7 (2)	P2—O3—Cd1	128.3 (4)
O6^iv^—Cd2—O10	177.1 (2)	P2—O3—Cd5^v^	111.1 (3)
O5—Cd3—O11	102.3 (2)	Cd1—O3—Cd5^v^	113.6 (3)
O5—Cd3—O2	81.4 (2)	P2—O3—Cd3^iii^	88.7 (3)
O11—Cd3—O2	89.5 (2)	Cd1—O3—Cd3^iii^	113.0 (2)
O5—Cd3—O7^iv^	106.1 (2)	Cd5^v^—O3—Cd3^iii^	93.3 (2)
O11—Cd3—O7^iv^	150.1 (2)	Cd5^vi^—O4—Cd2	118.2 (3)
O2—Cd3—O7^iv^	85.6 (2)	Cd5^vi^—O4—Cd1^x^	120.0 (3)
O5—Cd3—O1	98.6 (2)	Cd2—O4—Cd1^x^	99.3 (3)
O11—Cd3—O1	86.4 (2)	Cd4—O5—Cd3	110.2 (3)
O2—Cd3—O1	175.8 (2)	Cd4—O5—Cd2	106.7 (2)
O7^iv^—Cd3—O1	98.4 (2)	Cd3—O5—Cd2	120.2 (3)
O5—Cd3—O3^iv^	164.7 (2)	P1—O6—Cd2^iii^	128.6 (3)
O11—Cd3—O3^iv^	92.9 (2)	P1—O6—Cd1^xi^	109.3 (3)
O2—Cd3—O3^iv^	101.2 (2)	Cd2^iii^—O6—Cd1^xi^	94.1 (2)
O7^iv^—Cd3—O3^iv^	59.42 (19)	P1—O6—Cd4^v^	128.5 (3)
O1—Cd3—O3^iv^	79.8 (2)	Cd2^iii^—O6—Cd4^v^	90.9 (2)
O5—Cd4—O2^v^	166.4 (2)	Cd1^xi^—O6—Cd4^v^	97.2 (2)
O5—Cd4—O7^vi^	88.1 (2)	P2—O7—Cd4^v^	129.9 (4)
O2^v^—Cd4—O7^vi^	97.3 (2)	P2—O7—Cd3^iii^	99.9 (3)
O5—Cd4—O12	102.9 (2)	Cd4^v^—O7—Cd3^iii^	111.8 (3)
O2^v^—Cd4—O12	90.1 (2)	Cd2^x^—O8—Cd1	101.2 (3)
O7^vi^—Cd4—O12	83.8 (2)	Cd2^x^—O8—Cd1^x^	115.6 (3)
O5—Cd4—O10	78.0 (2)	Cd1—O8—Cd1^x^	133.6 (3)
O2^v^—Cd4—O10	93.3 (2)	P1—O9—Cd5^xii^	149.7 (4)
O7^vi^—Cd4—O10	159.4 (2)	P1—O9—Cd5	96.3 (3)
O12—Cd4—O10	113.9 (2)	Cd5^xii^—O9—Cd5	113.8 (3)
O5—Cd4—O6^vi^	86.1 (2)	P2—O10—Cd2	124.6 (3)
O2^v^—Cd4—O6^vi^	82.4 (2)	P2—O10—Cd4	113.0 (3)
O7^vi^—Cd4—O6^vi^	81.5 (2)	Cd2—O10—Cd4	96.7 (2)
O12—Cd4—O6^vi^	162.5 (2)	P2—O10—Cd1^x^	128.6 (3)
O10—Cd4—O6^vi^	82.4 (2)	Cd2—O10—Cd1^x^	89.6 (2)
O4^v^—Cd5—O9^vii^	87.0 (2)	Cd4—O10—Cd1^x^	97.1 (2)
O4^v^—Cd5—O3^vi^	168.8 (2)	P2^vi^—O11—Cd3	127.1 (4)
O9^vii^—Cd5—O3^vi^	82.7 (2)	P1—O12—Cd4	136.4 (3)
O4^v^—Cd5—O1^viii^	94.6 (2)	P1—O12—Cd5	94.7 (3)
O9^vii^—Cd5—O1^viii^	111.4 (2)	Cd4—O12—Cd5	121.2 (2)

Symmetry codes: (i) *x*−1/2, −*y*+1/2, −*z*+1; (ii) −*x*+1/2, −*y*, *z*+1/2; (iii) −*x*, *y*−1/2, −*z*+1/2; (iv) −*x*, *y*+1/2, −*z*+1/2; (v) −*x*+1, *y*−1/2, −*z*+1/2; (vi) −*x*+1, *y*+1/2, −*z*+1/2; (vii) *x*+1/2, −*y*+1/2, −*z*; (viii) *x*+1, *y*, *z*; (ix) *x*−1, *y*, *z*; (x) *x*+1/2, −*y*+1/2, −*z*+1; (xi) −*x*+1/2, −*y*, *z*−1/2; (xii) *x*−1/2, −*y*+1/2, −*z*.
